# Design Considerations in Developing a Text Messaging Program Aimed at Smoking Cessation

**DOI:** 10.2196/jmir.2061

**Published:** 2012-07-24

**Authors:** Michele L Ybarra, Jodi Summers Holtrop, A Tülay Bağci Bosi, Salih Emri

**Affiliations:** ^1^Center for Innovative Public Health ResearchSan Clemente, CAUnited States; ^2^Department of Family MedicineMichigan State UniversityLansing, MIUnited States; ^3^Department of Public HealthHacettepe UniversityAnkaraTurkey; ^4^Department of Chest DiseasesFaculty of MedicineHacettepe UniversityAnkaraTurkey

**Keywords:** Smoking cessation, mHealth, text messaging

## Abstract

**Background:**

Cell phone text messaging is gaining increasing recognition as an important tool that can be harnessed for prevention and intervention programs across a wide variety of health research applications. Despite the growing body of literature reporting positive outcomes, very little is available about the design decisions that scaffold the development of text messaging-based health interventions. What seems to be missing is documentation of the thought process of investigators in the initial stages of protocol and content development. This omission is of particular concern because many researchers seem to view text messaging as the intervention itself instead of simply a delivery mechanism. Certainly, aspects of this technology may increase participant engagement. Like other interventions, however, the content is a central driver of the behavior change.

**Objective:**

To address this noted gap in the literature, we discuss the protocol decisions and content development for SMS Turkey (or *Cebiniz birakin diyor *in Turkish), a smoking cessation text messaging program for adult smokers in Turkey.

**Methods:**

Content was developed in English and translated into Turkish. Efforts were made to ensure that the protocol and content were grounded in evidence-based smoking cessation theory, while also reflective of the cultural aspects of smoking and quitting in Turkey.

**Results:**

Methodological considerations included whether to provide cell phones and whether to reimburse participants for texting costs; whether to include supplementary intervention resources (eg, personal contact); and whether to utilize unidirectional versus bidirectional messaging. Program design considerations included how messages were tailored to the quitting curve and one’s smoking status after one’s quit date, the number of messages participants received per day, and over what period of time the intervention lasted.

**Conclusion:**

The content and methods of effective smoking cessation quitline programs were a useful guide in developing SMS Turkey. Proposed guidelines in developing text messaging-based behavior change programs are offered.

## Introduction

Cell phone text messaging is gaining increasing recognition as an important tool with a wide variety of health research applications. A search on the US Department of Health and Human Services’ Research Portfolio Online Reporting Tools (RePORT) website [1] using the keyword *text messaging *reveals over 40 studies currently funded that have a text messaging component. Surely, this is just a subset of the larger field of text messaging-based health programming, funded both privately and publicly, across the world, yet it demonstrates the public health field’s enthusiasm for this new technology.

Research studies have used text messaging as a data collection tool (eg, principal investigator [PI]: Boushey, 5U01CA130784 [2]; PI: Mundt, 1R43MH086152 [3]) and as a medication adherence enhancer (eg, PI: Belzer, 5U01HD040463 [4]). Perhaps the most interactive application of text messaging in the health arena is to change health behavior. Text messaging has been tested as the delivery mechanism for the main intervention content (eg, PI: Bull, 1R21MH083318 [5]; PI: Olson, 1R21HS018214 [6]) or for booster content that enhances content delivered online (eg, PI: Cornelius, 5R21NR011021 [7]). Data are still emerging and, at this stage, are preliminary. Nonetheless, reviews of the available literature find reason for optimism. A recent review reported 16 randomized controlled trials that involved text messaging, 10 of which reported significant improvement in their outcome measures; the remaining 6 reported positive trends [8]. Strong acceptability among intervention participants also was noted. Another recent review, focused specifically on health behavior change interventions, reported 9 sufficiently powered studies. Of these, 8 reported results supportive of a conclusion that text messaging can deliver content that affects behavior change [9].

Despite the growing body of literature documenting the outcomes of text messaging-based (sometimes also called mobile health or mHealth) interventions, very little is available about the development of these interventions. Owens and colleagues reported their experience developing a self-harm intervention to be delivered via text messaging. Based on feedback from service users and providers, the researchers chose a model whereby the participants created their own content, which then could be sent on demand in times of crisis [10]. Whittaker and colleagues reported the steps they followed to develop a multimedia smoking cessation program, including intensive focus group testing and pilot testing [11]. This latter study is a particularly useful guide in how to engage one’s user audience during the intervention development stage. What seems to be missing, however, are scientific papers that describe the practical decisions taken in developing the first drafts of the content of a text messaging program. As Cole-Lewis and Kershaw noted [9], many researchers seem to view text messaging as the intervention, yet it is simply the delivery mechanism. Certainly, there are benefits of text messaging communication that may increase the engagement and salience of the program content for participants. Like other interventions, however, it is the content, not the delivery mechanisms (eg, Internet, in-person), that is the central driver of behavior change.

## Methods

To address this noted gap in the literature, we describe the program development for SMS Turkey. Compared with the United States, where 23% of men and 18% of women are current smokers [12], an estimated 44% of men and 12% of women smoke daily in Turkey [13]. Despite Turkey’s high smoking prevalence rate, over half of smokers report a desire to quit, and 45% have made a quit attempt in the past year. To invigorate cessation rates, smoking cessation programs need to be easily accessible and have high reach. With an estimated 62 million cell phones in Turkey [14], there is sufficient reason for optimism about the feasibility of a text messaging-based smoking cessation program in this setting. We were funded by the US National Institutes of Health’s Fogarty International Center (R01TW007918) to develop and test SMS Turkey, a smoking cessation program delivered via text messaging. The program was designed in 2007–2008 and was created for adults seriously thinking about quitting smoking and living in Ankara, Turkey. Here, we describe the initial content and protocol development. Findings not only document the methodological development of SMS Turkey, but also provide direction for other researchers endeavoring to create mHealth behavior change programs.

## Results

### Methodological Considerations

Prior to developing the content, there are several methodological decisions to be made, many of which are determined by timeline and budget. One consideration is whether the program will include bidirectional messaging, whereby the participant provides input via text messaging that is then captured by the intervention software and responded to [9]. This can increase participants’ commitment to the study, and feelings that they are playing an active role in the intervention rather than passively receiving messages. It also can serve as an opportunity to tailor messaging or collect real-time data about the participant’s status (eg, current smoking behavior, level of cravings). On the other hand, the programming of the software to receive messages is much more complicated and therefore more costly and time intensive to develop than a unidirectional program that sends but does not receive and respond to participant messages. For pilot projects, it may be best to develop a unidirectional program and have research staff execute any bidirectional messaging through manual means. Once feasibility is determined, then a more complicated and costly program can be developed at the next stage. In the case of SMS Turkey, we chose to develop a unidirectional software program because of the intervention’s pilot nature.

Another important consideration is whether the research program will provide phones or text messaging plans for participants; or reimburse participants’ costs associated with receiving text messages. This decision should be based on the study goals. Some studies are aimed at harnessing the mobility of technology: for example, using cell phones to consistently reach unstable populations (eg, the marginally housed). In this case, it may be appropriate to give participants phones. In other studies, the motivation is to take advantage of the explosive increase in text messaging among adolescents and adults [15]. In this case, researchers need to be clear that the intervention is not intended for everyone (indeed, it is unlikely that there is one unique program that will address the needs and interests of all people at risk for a particular outcome), but rather for those who have adopted text messaging. In this case, the eligibility criteria should include having a text-capable phone, as well as an unlimited text messaging plan. If the target population is not using text messaging, then this mode may not be the best way to reach and engage the population.

In SMS Turkey, participants were required to have a cell phone and have used text messaging in the past year. It is free to receive text messages in Turkey; otherwise, we also would have required participants to be enrolled in an unlimited text messaging plan. If we had instead given participants cell phones or reimbursed them for costs associated with receiving program messages, we believe the findings had the potential to be adversely affected in three important ways. First, feedback from the participants about their study experience might not have reflected the views of the intended audience. Second, a study focus would necessarily have become management of the phones themselves. Third, the resulting data would have been less informative for potential scale-up of the intervention because agencies that might adopt the intervention are not likely to have resources to provide cell phones.

### Design Considerations

Although text messaging interventions are relatively new, effective interventions using other modalities likely exist that can be used to guide the design. In smoking cessation, quitlines (ie, cessation counseling delivered via telephone) are widely available and known to be an effective method of counseling that reaches many smokers [16-18]. Quitlines were an especially amenable guide for SMS Turkey because they are grounded in behavior change theory and use ongoing contact between an interventionist (phone counselor) and participant (smoker), similar to how participants may interact with the information received through the text messages in an ongoing fashion to affect behavior change. Also, the proactive nature of the counselor calling the participant is more similar to text messaging-based communication compared with the reactive nature of most Internet-based interventions that rely on participants to log on to a website for information [19].

Another early decision that needs to be made is the overall study length: over what period of time will messages be sent? The intervention needs to be long enough to affect behavior change without being so long as to cause participants to lose interest and drop out. SMS Turkey was developed to be a 6-week program: 2 weeks of prequit and 4 weeks of postquit messages. This was based on the length of most quitline programs [20-22] and on successful implementation of a text messaging-based smoking cessation program in New Zealand of similar length [23]. Future interventions may explore longer program periods.

Another decision is the number of messages that will be sent per day or per week. This should be based on the target population (eg, how many messages do they receive in a typical day?) and on the literature associated with the target behavior. Similar to the overall intervention length, it is important to deliver a sufficient intensity of messages to affect behavior change, while not overwhelming participants to the point where they no longer read the messages. Our development survey suggested that almost half of adult smokers in our target population texted daily [24]. Therefore, we felt comfortable that a daily schedule that fluctuated based on the quitting curve would be appropriate. For smoking cessation, telephone-based programs’ sessions increase in frequency around the quit date, and then gradually reduce in frequency as the participant gets further along the relapse curve associated with cessation [25]. We tried to create a similar experience in SMS Turkey: for the first 2 weeks leading into cessation, participants received three messages per day. As participants got closer to the quit day, we sent five messages per day. On the quit day and the following day, we sent eight messages each day. For the next 2 days, six messages were sent; this was reduced to five messages on the next day. We sent four messages on each of the last 2 days of their first postquit week. For the next 2 weeks, participants received two messages per day. In the final week, messages were pared down to one message per day.

Decisions about tailoring also need to be made at this stage. Tailoring uses information that an individual has provided about his or her circumstances to personalize the information that the participant receives to affect behavior change [26]. Tailoring increases the self-relevance of material, thereby increasing the likelihood that participants will be motivated to act on the material [27-30]. A recent review of computer-based health behavior change interventions suggests that the more dynamically the program is tailored, the stronger the efficacy data are likely to be [31]. Each point of tailoring results in more content needing to be written, however, so the number of points the program is tailored on should be weighed against the amount of time and budget the research team has. For researchers who are interested in concrete examples of how tailoring may be applicable to their program, Strecher and colleagues provide useful direction on their website [32].

In SMS Turkey, we chose to create different content paths for participants based on their progress along the quitting curve [16,21]. Previous data suggest that most smokers who relapse will do so within the first 2 days after quitting; at 7 days, the relapse curve begins to bottom out [25]. Therefore, we created paths for participants who were quit 2 days after quit day versus those who were smoking; and for those who were quit 7 days after quit day versus those who were smoking. The pathing could be done manually by research staff or automatically by the software program using a bidirectional data collection and response system. Due to time and financial constraints in the pilot study, research staff contacted participants at each time point and then manually pathed the participant to the applicable messages based on his or her response.

Researchers also will need to decide whether the program will have unique messages throughout the program or whether some messages will be repeated. This is particularly relevant if the intervention has a relapse path for those who are unsuccessful in enacting the targeted behavior change: do these participants repeat the previous content, or do they receive a different set of messages? In SMS Turkey, we decided to create unique messages such that participants would receive new messages across the program and paths.

A last decision is whether to enhance the text messaging program with other outreach efforts: this might include telephone calls from a counselor or an interactive website that reinforces important concepts. The interactive website option may be particularly important if the intervention is trying to demonstrate a new skill, such as how to use a condom in a sexual health intervention. The potential benefits need to be weighed against potential costs and how additional components may affect the potential scale-up of the program. Given the resource-limited setting in Turkey, we wanted to develop a stand-alone program that did not require the funding of in-person support. Because comparatively fewer people had access to the Internet than to text messaging [24], we also decided that an online add-on would not be useful. Therefore, we decided that the SMS Turkey program would rely solely on the text messaging content.

### Drafting the Content

The content should be guided by experts in the field and grounded in a theoretical model. Ideally too, formative research should be conducted within the target population to identify any unique challenges they face, as well as to gather feedback about the content as it is being developed. In SMS Turkey, we conducted a quantitative survey among 150 adult smokers living in Ankara to better understand the technology use and smoking experiences of adults who were seriously thinking about quitting smoking in the next 30 days and living in Turkey’s capital city [24].

Smoking cessation quitlines rely heavily on cognitive behavioral therapy [33,34]. Cognitive behavioral therapy focuses on altering the individual’s way of thinking (cognitive processes) and acting (behavioral actions). Components include identifying new behaviors to be substituted for smoking-related activities, making a commitment to quitting, considering consequences of continued smoking, seeking information about smoking, controlling cues that may trigger the urge to smoke, and rewarding oneself for not smoking [35]. Additional program components include self-efficacy [36-39] (eg, reinforcing beliefs that the person is capable of quitting, while also teaching the person how to set goals to master quitting) and relapse prevention [40-45] (eg, helping participants identify potential triggers and coping strategies). Smoking cessation guidelines also recommend a combination of pharmacotherapy and behavioral strategies [33,46,47]. In SMS Turkey, the content provided cognitive and behavioral strategies. Instead of also providing pharmacotherapy, however, the program encouraged participants who smoked 10 or more cigarettes a day to separately talk with their health care provider.

Once the literature has been consulted and formative research conducted, a content map needs to be drafted. For SMS Turkey, five stages of messages emerged from the quantitative survey, a review of the literature, and the research team’s clinical experiences [16,17,24,25,41,42,48-50]. The content aim for each stage was as follows. (1) *Prequit messages*: clarify reasons for quitting; understand smoking patterns and tempting situations, triggers, and urges; practice altering smoking patterns; learn about pharmacotherapy options and obtain prescription or medication; prepare to quit (eg, preparing environment, seeking support). (2) *Early quit*: identify common difficulties and discomforts (eg, what to expect and how to deal with them); emphasize use of coping strategies and to not over-think the decision to quit; emphasize self-identified reasons for and benefits of quitting; manage issues and side effects of medication; increasing pleasurable and other activities. (3) *Late quit*: become vigilant to recognize relapse in a different way (eg, situations, confidence); learn how to deal with issues that arise as a nonsmoker (eg, handle stress, moods); learn about weight gain and preventing weight gain; learn to reward and care for oneself; learn how to think of oneself as a nonsmoker; learn the benefits of a nonsmoking lifestyle. (4) *Slip/relapse*: know that many slip and how to get back on track; clarify reasons for quitting and recommit; learn about what didn’t work and new strategies. (5) *Encouragement*: encourage those unable to quit with this attempt to try quitting again in the future; learn the norms of quitting and the quitting process.

**Figure 1:**
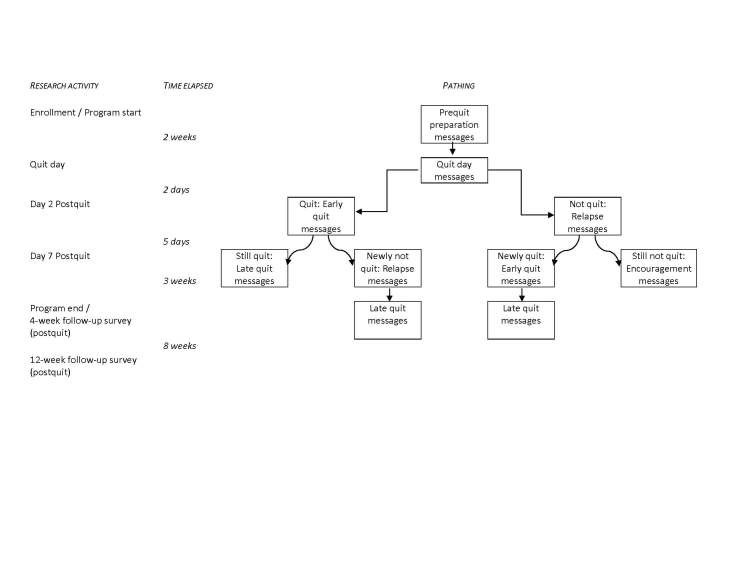
SMS Turkey text messaging program flow.

As shown in Figure 1, all participants received the prequit and early quit messages. According to our tailoring plan, at day 2 postquit, participants were pathed to more early quit messages if they continued to be quit, or pathed into slip/relapse messages if they were smoking. Participants who spontaneously shared that they had tried but just cannot quit at day 2 were given the option to opt-out and end the program immediately, or to be pathed to the Encouragement arm. Content was tailored again at day 7. Participants who were quit at day 2 and continued to be quit at day 7 moved into late quit messages; participants who were quit at day 2 but were then smoking at day 7 were moved into slip/relapse and then moved into late quit; those who were smoking at both day 2 and day 7 were moved into encouragement messages. We included this last, abbreviated arm for people who continued to struggle with quitting in order to be respectful of the participant’s individual quitting processes. If participants are finding it extremely difficult to quit, sending them text messages about how well they are doing with quitting, or even how they should “not quit quitting” might be perceived as shaming and may disenfranchise them during what might be a teachable moment. Instead, we tried to capitalize on this opportunity by helping participants frame their quitting attempt as a positive step forward in the sometimes long quitting process.

As shown in Table 1, we crafted eight different message types. Participants need to receive messages that provide specific and actionable strategies to cope with the discomfort of quitting. It also is important to educate smokers about the benefits of quitting to motivate them through difficult periods. We created an algorithm for each message type across each of the program stages (see Table 2). Armed with this guide for message creation, we produced a draft and then iterated the program content.

**Table 1:** Quit message types.

Message type	Description
Preparing to quit	Describes steps to take in preparing to quit smoking, including preparing oneself mentally and physically, and preparing one’s own and others’ environment
Benefits of quitting	Describes the health-related, social, and financial benefits of becoming a nonsmoker
Coping and coping strategies	Describes and encourages the effectiveness and use of cognitive and behavioral strategies to avoid smoking during a craving or impulse to smoke
Discomfort and difficulties	Discusses discomfort associated with the quitting process and how the participant may see his or her discomfort as normal and how to cope with such discomfort
Encouragement	Offers motivation and support to the participant to continue with quitting
Nicotine replacement therapy and pharmacotherapy	Encourages consideration and proper use of pharmacotherapy aids for use with the program in quitting smoking, such as nicotine replacement and other medications
Quitting skills	Teaches specific skills to aid in the quitting process
Relapse	Discusses the norms of slipping and how to get back on track; Clarifies reasons for quitting and to recommit; Teaches the participant to learn about what didn't work and new strategies

**Table 2:** Algorithm for cessation messages.

Message type	Number of messages per quitting stage					
	Prequit	Quit day and day 2	Early quit	Late quit	Relapse	Encouragement to try quitting again later
Preparing to quit	17	0	0	0	0	0
Benefits of quitting	4	2	3	19	3	2
Coping and coping strategies	14	4	11	6	7	1
Discomfort and difficulties	1	6	3	1	2	0
Encouragement	3	2	5	3	5	2
Nicotine replacement therapy and pharmacotherapy	3	2	2	1	2	1
Quitting skills	2	0	0	6	0	0
Relapse	0	0	0	0	5	0

We developed the content using the following guidelines.

#### Guideline 1

Write the messages to flow like a discussion across the day. For example, in the prequit stage, we encouraged people to start a smoking diary. The following are three messages that followed each other consecutively across the day:

Message 1: *When and why do you smoke? Start a smoking diary. Keep track of when you smoke, what you’re doing (the activity), how you’re feeling, and your craving (from 1 to 3).*

Message 2: *Write down a list of reasons why you want to quit smoking. Put it where you can see it.*

Message 3: *How is your smoking diary going? Put the “diary” paper on your cigarette pack with a rubber band. Every time you have a cigarette, fill out 1 line.*

The content of each new message builds on the previous message just as a conversation would.

Similarly, messages across days can refer to each other. For example, in the prequit stage, we tell participants to make a plan to reward themselves for quitting:

Write down a list of rewards for yourself—plan what you are going to do for yourself after you’ve quit for 1 week, for 2 weeks, for a whole month.

Two days before their 1-week quit anniversary, participants receive the following message:

Have you been rewarding yourself for not smoking? Look back at your Rewards List—what did you write down on your list for your 1-week special reward? Too fun!

#### Guideline 2

In the initial stages of message creation, the number of characters is not important. Certainly, brevity is the aim, but it is not useful to keep vigilant and stay below 160 characters (the limit for one text message), as the content itself is changing. This can be the last step before finalizing the content.

#### Guideline 3

Color-coding the messages in a spreadsheet program provides a useful visual cue to ensure that the message types are well spaced across days.

### A Example of the Finished Product

To provide a concrete example of a finalized pool of text messages that are ready for subsequent testing, we show messages in the encouragement path in Table 3.

**Table 3:** Example of SMS Turkey content: the encouragement path.

Timing	Message text
Day 1a	Most smokers try to quit 6–7 times before they quit for good. Don’t quit quitting!
Day 1b	It’s a great thing that you’ve tried to quit smoking. You learned some things that you can apply to the next time you try to quit. What worked? What didn’t?
Day 2a	Quitting smoking is the single most important step you can take to improve your health.
Day 2b	Medicines that treat craving can double your success. Try medicine next time you quit. If you used medicine, try a different one next time. Ask your doctor.
Day 3a	Smokers live an average of 7–12 years less than nonsmokers. Consider quitting again!
Day 3b	Whatever you decide about smoking, believe in yourself. You CAN quit smoking if you put your mind to it and have a plan for success.

### Cultural and Cost Considerations

As mHealth research begins to extend beyond developed countries, cultural considerations in designing content and programming should be taken into account. The team leading the content development resided in the United States. The messages, therefore, were originally written in English. To ensure that the messages were culturally relevant and salient, a bilingual person translated the SMS Turkey messages from English into Turkish, paying particular attention to capturing cultural meaning and context. Then, a separate team member back-translated the messages into English. This typical procedure for translation and back-translation helps to ensure that the essence of the content is captured in the translation. The messages also were reviewed by students at Hacettepe University for understandability and credibility.

 As noted above, a consideration during the protocol development stage is whether and how to reimburse costs that the participant may incur as a result of participating in the intervention. In Turkey, and most countries aside from the United States, receiving text messages is free. Thus, participants can take part in the program and receive as many text messages as possible without incurring a cost. As such, text messaging-based health programs are as free to participants as web-based health programs in countries such as Turkey.

## Discussion

As text messaging becomes more common in research and intervention delivery, it will be important to document not only outcomes, but also content and protocol development procedures. This is particularly true as researchers struggle to see text messaging as the delivery mechanism, rather than the intervention itself [9]. For this reason, this paper focuses specifically on the development plan of SMS Turkey (called *Cebiniz birakin diyor *in Turkey).

Program development includes careful attention to not only the theoretical underpinnings of the behavior change strategy and associated content, but also the design, including how messages will be tailored, how participants may be pathed to different content based on their smoking status, and the number of messages participants will receive per day. The program must also attend to the technology use of the target population by including elements that fit that group. If the target population is not using text messaging, alternative delivery modes should be considered instead.

Effective health behavior change programs are guided by strong theoretical models [20,51,52]. Recent reviews have suggested, however, that many available text messaging-based behavior change programs may not be using theoretically based intervention strategies that have proven utility in producing behavior change [53]. Even though the technology is new, existing programs developed for more traditional environments can likely serve as an applicable guide when designing the content. In this study, we found that the theory and protocols underpinning telephone-based smoking cessation programs were useful guides for developing similar SMS Turkey program components. Indeed, this previous work in the field of smoking cessation informed the SMS Turkey content map, which guided the tone of the messages, and stage (eg, prequit versus early quit) and type of messages (eg, coping strategies, relapse) delivered over the course of the program and the participant’s quit progress.

Although the intervention context behavior was smoking cessation, this development strategy is generalizable to other text messaging-based behavior change interventions. It should be noted that, as with many pilot projects, budget and timeline restrictions limited exploring all possible aspects of program development and tailoring. Certainly too, although we used theoretical models in developing the intervention, we based the program more generally on the key areas of behavior change found to be effective in quitline counseling. Our intent was to adapt successful quitline interventions to this new modality rather than start anew and develop a completely new theoretical perspective.

## Abbreviations

PI: principal investigator
